# In vivo precision of digital static interocclusal registration for full arch and quadrant arch scans: a randomized controlled clinical trial

**DOI:** 10.1186/s12903-022-02612-5

**Published:** 2022-12-01

**Authors:** Noha Morsy, Mohammed El Kateb

**Affiliations:** Department of Conservative Dentistry, Faculty of Dentistry, Alexandria University, Alexandria, Egypt

**Keywords:** Digital, Full arch, Quadrant, Static interocclusal record

## Abstract

**Background:**

Clinical studies comparing the accuracy of digital and conventional records for static interocclusal registration are lacking. Therefore, the purpose of this clinical study was to assess the precision of digital interocclusal registration compared to conventional registration for full arch and quadrant arch conditions.

**Methods:**

Nine individuals with complete natural dentition were enrolled in this study. Each participant received digital scans, conventional impressions, and static interocclusal records according to the following groups: group DF, full arch digital scans and bilateral buccal scans with Medit i700 intraoral scanner (IOS); group DQ, quadrant arch digital scans and unilateral buccal scans with Medit i700 IOS; group CF, full arch polyvinyl siloxane (PVS) impressions and PVS interocclusal records; group CQ, quadrant arch PVS impressions and PVS interocclusal records. For group CF and group CQ, the impressions were poured, mounted with the silicone bites, scanned with a laboratory scanner, and articulated virtually with buccal scans with the laboratory scanner. In each group, each participant received three interocclusal records to repeat the virtual articulation three times and the articulated models were saved as STL files. The STL files were imported into a 3D-processing software to calculate the discrepancies between repeated measures using best-fit-alignment method. The significance between the study groups was calculated with two-tailed paired t-test at *P* < 0.05.

**Results:**

For full arch, group DF showed significantly better precision with a mean value of 31 ± 19 µm compared to 204 ± 81 µm for group CF (*P* = 0.0003). Similarly, for quadrant arch, group DQ showed significantly better precision with a mean value of 18 ± 6 µm compared to 255 ± 136 µm for group CQ (*P* = 0.0009). No significant difference in precision was found between quadrant arch and full arch when the digital or the conventional method was used.

**Conclusions:**

The digital approach had significantly better precision for static interocclusal registration compared to the conventional method in both full and quadrant arch situations.

*Trial Registry* This clinical trial was registered on 06/07/2022 in the Pan African Clinical Trial Registry database, the number for the registry is PACTR202207648490275.

## Background

An accurate static interocclusal registration is necessary for occlusal analysis and fabrication of a successful fixed restoration. Registration of static interocclusal relationship can be performed either by using conventional or digital methods. The conventional method comprises a physical bite record in maximum intercuspal position (MIP), mounting of stone casts with the physical bite, and buccal scanning of the mounted casts with a laboratory scanner to perform the virtual articulation. In the digital method, the dental arches are scanned with an intraoral scanner (IOS) to obtain virtual models. A virtual interocclusal record (VIR) is obtained by scanning the buccal surfaces of teeth in MIP with the IOS. The virtual models of dental arches and the VIR are finally aligned with the IOS software to complete the virtual articulation [1, 2].

In daily clinical practice, time and effort saving, and patient comfort and satisfaction are paramount as long as the procedure can be achieved efficiently and precisely. Consequently, taking a quadrant impression is preferred by many practitioners when possible either in conventional or digital workflow. Moreover, the complete digital workflow is becoming more popular for the same reasons [3]. The virtual articulation involves best-fit-alignment of virtual models and buccal scan images by using areas of occlusal contacts as reference points [4]. Similarly, precise articulation of stone casts with a physical bite must ensure vertical and horizontal stability of mounted casts through adequate occlusal stops [5]. To the author**s'** knowledge, only three studies [6–8] compared the accuracy of static interocclusal registration with conventional and digital methods, and reported better results for the digital approach. However, there are no published clinical studies that compared conventional and digital articulation for full arch cases. Moreover, only two studies [1, 3] compared the accuracy of articulation between full arches and quadrant arches and the results were contradictory. Arslan et al. [3] recommended full arch scan over quadrant scan to provide more occlusal landmarks and enhance the articulation accuracy. On the contrary, Edher et al. [1] reported a better accuracy for VIRs in case of quadrant scans compared to full arch scans. They attributed this finding to the tilting effect toward the VIR side that occurs during the virtual articulation of full arch scans.

Therefore, this clinical study was conducted to assess the precision of digital interocclusal registration compared to conventional registration for full arch and quadrant arch conditions. The null hypothesis was that no difference would be found in precision between digital and conventional methods for both full arch and quadrant arch conditions.

## Methods

### Study design, sample size, and patients selection

This study was conducted as a randomized double-blinded controlled cross-over clinical trial with within-subject comparison. The study followed the Consolidated Standards of Reporting Trials guidelines (CONSORT) and The Code of Ethics of the World Medical Association (Declaration of Helsinki) for experiments involving humans [9]. The study received the approval of the scientific research ethics committee at the Faculty of Dentistry, Alexandria University, Alexandria, Egypt (IRB NO 0460-06/2022, IORG 0008839). The study was registered in the Pan African Clinical Trial Registry (ID: PACTR202207648490275) on 06/07/2022. The sample size was calculated based on the results of Iwauchi et al. [6] by using a software program (G*power 3.1.9.6; Heinrich-Heine-Universität, Germany), assuming an alpha error of 5%, a study power of 80%, and an effect size of 0.92, a minimum sample size of n = 9 per group was needed [10]. The included participants were selected from outpatient’s clinic at Conservative Dentistry Department, Faculty of Dentistry, Alexandria University. The selected participants aged between 25 and 35 years with complete natural dentition, skeletal angle class I, and good oral and general health. Individuals with abnormal occlusion, periodontitis, or orofacial or dental pathology were not eligible for the study [6]. All selected participants provided informed consent.

### Clinical procedures

All clinical procedures were performed by a single experienced practitioner under controlled environmental conditions. Each participant received conventional and digital impressions and interocclusal records according to a randomly allocated sequence using closed envelopes according to a computer-generated list by an independent operator as patients and examiners were blinded to the allocation sequence.

### Full arch digital impressions and virtual interocclusal records (group DF)

First, the participants were trained to occlude in MIP with a consistent occlusal force. For group DF, each patient received a full arch digital scan for the mandible and maxilla with Medit i700 IOS (Medit Corp., Seoul, South Korea) according to the manufacturer instructions. To articulate the virtual models, the participant was asked to close as trained in MIP and bilateral posterior buccal scans were obtained with the IOS. Each buccal scan was 36 mm wide and 15 mm high and included two or three teeth [11]. The articulated models were saved as STL file. The maxillary and mandibular full arch scans were cloned using the IOS software (Medit Link, Medit Corp., Seoul, South Korea), the first VIR was deleted, and a new VIR was captured with the IOS in MIP to rearticulate the same scans with a second VIR and the articulated virtual models were saved as STL file. The same process was repeated with a third VIR and the rearticulated models were saved as STL file so that for each participant, three virtual interocclusal records were obtained to articulate the same maxillary and mandibular full arch digital scans and the virtually articulated models were saved as three STL files.

### Quadrant arch digital impressions and virtual interocclusal records (group DQ)

For group DQ, for each participant, a quadrant arch scan was taken for the maxilla and mandible with the same IOS, a unilateral buccal scan was obtained with the patient occluding as trained in MIP, and the virtual models were articulated and saved as STL file. The same procedures were followed as in group DF and the virtual articulation was repeated three times for each patient with the same quadrant scans and the articulated models were saved as three STL files.

### Full arch conventional impressions and silicone interocclusal records (group CF)

For group CF, each patient received a maxillary and a mandibular complete arch conventional impression with polyvinyl siloxane (PVS) impression material (Imprint4, 3 M ESPE, Seefeld, Germany) in stock trays (New IN Toothed All Jaw Tray, Dentsply Sirona, NC, USA) according to the manufacturer instructions. The impressions were poured in type IV plaster (Fujirock, GC Europe, Leuven, Belgium). Three interocclusal records were made for each patient with PVS (Futar D, Kettenbach GmbH, Eschenburg, Germany) in MIP. The stone models were scanned with a laboratory scanner (Vinyl, Smartoptics Gr, Oslo, Norway). The casts were hand articulated with the first silicone interocclusal record and fixed with sticky wax then buccal scanning was performed with the laboratory scanner to articulate the virtual models and save the articulated models as STL file using the laboratory scanner software (Exocad, Exocad GmbH, Darmstadt, Germany). The maxillary and mandibular scans were cloned with the laboratory scanner software and the first buccal scan image was deleted. The stone models were hand articulated using the second silicone bite and fixed with sticky wax, a new buccal scan was captured for the articulated stone casts with the laboratory scanner to rearticulate the virtual models and save them as STL file. The same process was repeated for the third silicone bite so that for each participant the same scans for maxillary and mandibular stone models were articulated three times with the three silicone records and the virtually articulated full arch models were saved as three STL files.

### Quadrant arch conventional impressions and silicone interocclusal records (group QF)

For group CQ, the clinical procedures were the same as in group CF except that quadrant arch conventional impressions were taken with quadrant trays (Net Tray Premium for Local Teeth, YDM, Tokyo, Japan), for each patient three virtually articulated quadrant arch models were obtained and saved as three STL files.

### Assessment of precision

The STL files of the articulated models of each study group were numbered sequentially according to a computer-generated list and the assessment was conducted by a single blinded examiner. The STL files were imported into a 3D-processing software (MeshLab, version 2016.12, National Research Council, Pisa, Italy) to evaluate the precision. Each pair of STL files were superimposed against each other, for each participant, three superimpositions were obtained in each study group using the software best-fit-alignment algorithm tool. The software aligned each pair of STL files by selecting 50,000 random data points on the first dataset (STL file) and finding the closest points on the other dataset. Moreover, a second superimposition was carried out using 100,000 data points to obtain more accurate best-fit-alignments. The precision was measured by calculating the Hausdorff distance and Root Mean Square (RMS) error values with the software. The Hausdorff distance is the longest distance between each point on one STL file and the closest point on the other STL file in x, y, and z axes. The Hausdorff distance indicates the 3D spatial divergences between each pair of STL files. RMS is a mathematical tool that represent the deviation of the superimposed STL files from having the best-fit. The lower RMS error indicates less deviation between repeated measures and consequently better precision [12]. RMS error values were calculated by the software to indicate the precision of each study participant and presented in Table 1. The software provided a color-coded mapping for qualitative assessment of deviations between 3D datasets showing the distribution and magnitude of the deviations. The color map included green and orange regions indicating deviations between 100 and 760 µm and blue regions indicating deviations between 0 and 100 µm.

**Table 1:** Precision (µm) of conventional and digital interocclusal records for full arch and quadrant arch in each participant

Participant	Group DF			Group DQ			Group CF			Group CQ		
	Mean	SD	Median	Mean	SD	Median	Mean	SD	Median	Mean	SD	Median
1	30	16	38	17	3	17	213	13	213	311	57	328
2	24	11	22	12	4	11	267	92	309	484	340	286
3	72	6	70	20	0.5	20	273	20	278	205	80	166
4	40	22	48	30	8	30	133	13	126	116	12	113
5	15	7	16	15	2	15	312	146	354	190	58	210
6	17	7	20	11	1	10	218	91	244	197	44	217
7	47	7	46	16	2	17	56	19	60	60	12	65
8	13	2	12	19	17	9	226	4	226	361	25	352
9	22	5	19	24	4	22	136	46	159	374	103	355

*SD* Standard deviation

### Statistical analysis

RMS values were exported to a spreadsheet (Microsoft Excel 2019 VL 16.44, Microsoft Corp., WA, USA). The mean, median, and standard deviation were calculated for each participant. For each study group, the mean, median, standard deviation, and 95% confidence interval were calculated using the mean values of the nine participants. Two-tailed paired t-test was used to evaluate the significant difference between each pair of the study groups at *P* < 0.05.

## Results

**Fig. 1:**
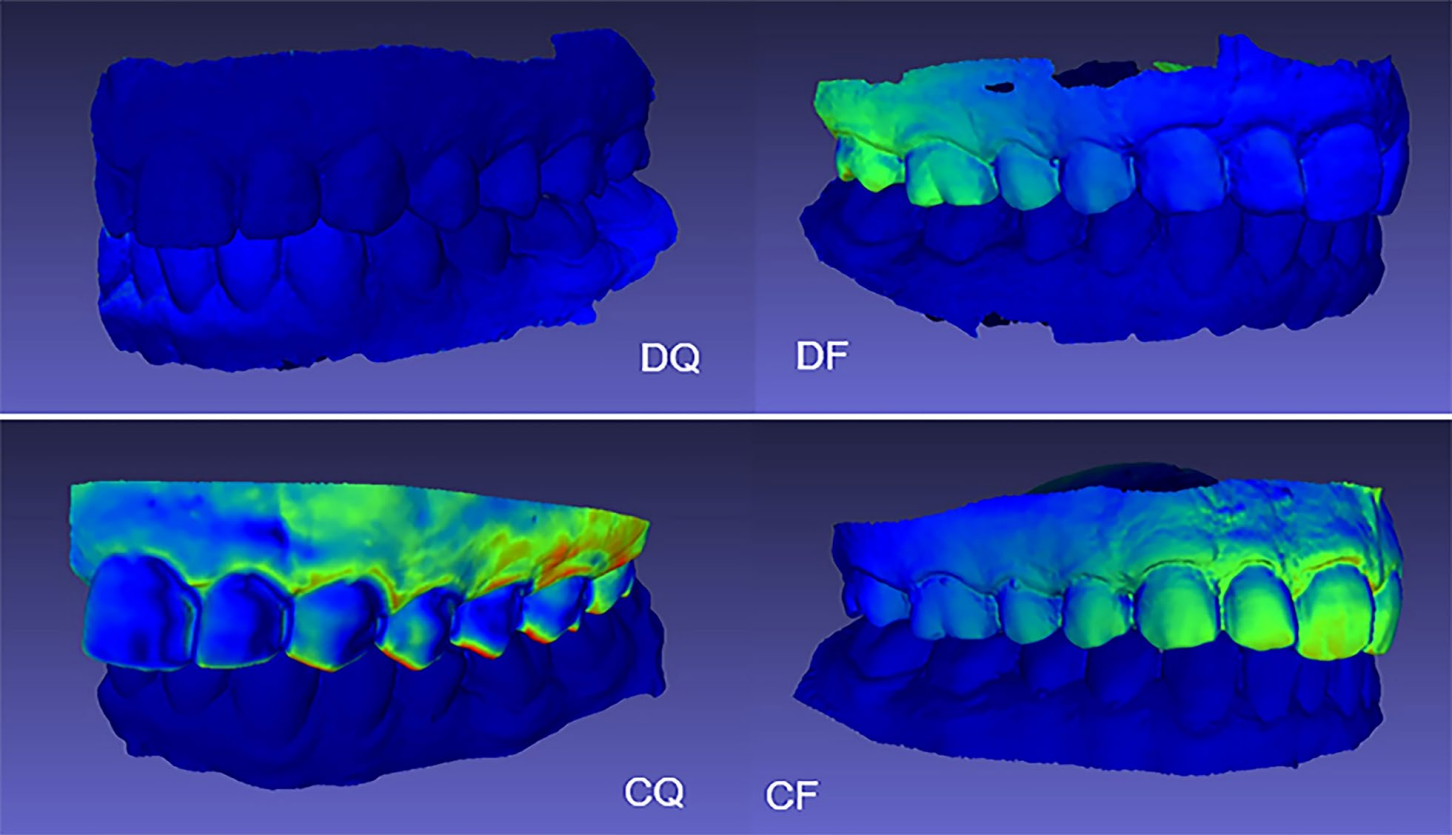
Representative examples for color mapping of discrepancies between 3D datasets of a participant in the four study groups. Conventional method (group CF and CQ) showed greater discrepancies (shown in green and orange) while digital method (group DF and group DQ) showed less discrepancies (shown in blue)

The mean, median, and standard deviation for each participant are summarized in Table 1. The mean, median, standard deviation, and 95% confidence interval for the study groups are displayed in Table 2. All study groups showed acceptable precision mean values of less than 500 µm according to the American Board of Orthodontics (ABO) objective grading system [13]. For full arch, the digital method (group DF) showed significantly better precision with a mean value of 31 ± 19 µm compared to 204 ± 81 µm for the conventional method (group CF) (*P* = 0.0003). Similarly, for quadrant arch, the digital method (group DQ) showed significantly better precision with a mean value of 18 ± 6 µm compared to 255 ± 136 µm for the conventional method (group CQ) (*P* = 0.0009). No significant difference in precision was found between quadrant arch and full arch groups when digital or conventional methods were used (*P* > 0.05). Figure 1 shows representative examples for color mapping of discrepancies between 3D datasets of a participant in the four study groups. Qualitative analysis of color mapping showed greater discrepancies (regions shown in green and orange) with conventional method compared to digital method (regions shown in blue). In addition, localized areas of significant deviations above 500 µm were found in group CF and group CQ only, such deviations were not detected in the digital groups.

**Table 2:** Precision (µm) of conventional and digital interocclusal records for full arch and quadrant arch

Arch area	Digital record				Conventional record				*P* value
	Mean	SD	Median	95%CI	Mean	SD	Median	95%CI	
Full arch	31	19	23	19/44	204	81	218	151/257	0.0003*
Quadrant arch	18	6	17	14/22	255	136	205	166/344	0.0009*
*p* value	0.0688				0.2560				

*CI* Confidence interval, *SD* Standard deviation

*Significant difference at *P* value <  0.05

## Discussion

This study aimed to compare the precision of static interocclusal registration with physical silicone records and IOS for both quadrant arch and full arch conditions. The null hypothesis was rejected as the VIR with IOS produced significantly more precise static articulation compared to the silicone bite in both full arch and quadrant arch conditions. When a silicone bite is used for stone casts mounting, inaccuracy of articulation can result from the dimensional changes in the silicone, inaccurate seating of the stone casts into the bite, and compressibility of the silicone bite [14]. Moreover, when the indirect digital workflow is applied as in current research, the mounted casts with a silicone bite are scanned to convert the physical articulation into virtual articulation, and errors can result during the scanning process. During scanning a pair of mounted casts, which are heavier and larger than a single cast, the laboratory scanner rotates, translates, and tilts the casts, this process can introduce displacement of the articulated pair of casts and change the registered MIP record obtained from the patient [2, 15].

In this research, a precision mean value less than 500 µm was considered acceptable according to the ABO objective grading system [13]. A deviation up to 500 µm in articulation of casts for fixed prosthodontics can be considered significant as the tolerance for articulation in fixed prosthodontics is much smaller than for orthodontics. However, the threshold value stated by the ABO system was the only available data in the literature to compare the results of this study.

The methodology applied in this research to measure the precision was used in previous studies evaluating the reproducibility of VIRs [6, 7, 16, 17]. This methodology depends on calculating the distance between two points on a pair of datasets in x, y, and z axes to measure the 3D spatial discrepancies [12]. In the current research, the authors focused on the precision of the interocclusal records, as a result the digital scans and conventional impressions for each patient were not repeated, only the interocclusal records, consequently any deviations detected by the 3D processing software between the repeated articulations were entirely related to a positional deviation of articulated models or an imprecise articulation and not affected by any dimensional changes that could have been occurred if the impression, stone models, or digital scans have been repeated.

The findings of this clinical study are in accordance with Iwauchi et al. [6] and Ries et al. [7] who reported that VIR is more precise than conventional interocclusal record. In addition, the results of this study agree with Camcı et al. [18] and Ayuso-Montero et al. [19] who reported better precision for VIR with IOS in detecting occlusal contact area compared to conventional interocclusal record. Similarly, a study by Gjelvold et al. [20] reported that VIR can produce crowns with better occlusion than conventional record. However, the results of this study disagree with Iwaki et al. [8]. The differences in the results may be attributed to the settings of the experiments.

The purpose of this clinical study was to help the practitioners when taking clinical decisions on whether to shift from conventional to digital bite record or not. The results of this study were in favor of digital bite registration to gain more precise virtual articulation. In addition, the findings of the current study can help the clinician when dealing with a case that could be taken with a quadrant scan or impression to save time and effort while the precision is maintained or even improved. In this study, the quadrant arch did not differ significantly in precision from the full arch when conventional or digital approach was adopted. Moreover, when the optical bite was used to articulate quadrant scans (group DQ) the precision was higher than in full arch scans (group DF). This agrees with Edher et al. [1] who reported a better accuracy for virtual interocclusal records (VIRs) in case of quadrant scans compared to full arch scans. They attributed this finding to the tilting effect toward the VIR side that occurs during the virtual articulation of full arch scans. Another possible explanation for this finding is that in quadrant arch there are fewer virtual images to align and stitch and consequently less discrepancy [21]. Based on the results of this clinical research, the clinician is encouraged to use quadrant scans and virtual interocclusal records for the best precision with less time and effort in clinical situations that do not necessitate a full arch scan or a complete digital workflow in full arch cases for more precise static articulation.

The limitations of this study included that only one laboratory scanner and one IOS were used for the scans and the virtual articulation. In addition, the accuracy was assessed based on the precision only. However, trueness was not possible to be calculated in this research as the study was conducted in vivo where obtaining a reference standard was impossible. Further studies are recommended to assess the reproducibility of different IOSs for VIRs. Moreover, further research is required to evaluate the VIRs accuracy for dynamic interocclusal registration.

## Conclusion

Within the limitations of this study, it was concluded that virtual interocclusal records in MIP with IOS had significantly better precision compared to conventional interocclusal records for both quadrant arches and full arches.

## Data Availability

The datasets used and/or analysed during the current study are available from the corresponding author on reasonable request.

## Abbreviations

CI: Confidence interval
CONSORT: Consolidated Standards of Reporting Trials guidelines
3D: 3 Dimensional
IOS: Intraoral scanner
MIP: Maximum intercuspal position
PVS: Polyvinyl siloxane
RMS: Root mean square
SD: Standard deviation
VIR: Virtual interocclusal record
